# Intersubject variability in a comprehensive numerical assessment of operator electromagnetic exposure to TMS

**DOI:** 10.3389/fpubh.2025.1705893

**Published:** 2026-02-02

**Authors:** S. D’Agostino, M. Colella, R. Falsaperla, M. Liberti, F. Apollonio

**Affiliations:** 1 Department of Information Engineering, Electronics and Telecommunications, Sapienza University of Rome, Rome, Italy; 2 Department of Occupational and Environmental Medicine, Epidemiology and Hygiene, Italian National Institute for Insurance Against Accidents at Work, Rome, Italy

**Keywords:** human variability, intersubject comparison, numerical dosimetry, occupational exposure assessment, transcranial magnetic stimulation

## Abstract

**Introduction:**

Transcranial magnetic stimulation (TMS) is increasingly used in clinical and research settings, often requiring the operator to remain in close proximity to the stimulation coil. While regulatory guidelines exist for patient safety, the workplace exposure assessment, particularly its dependence on operator anatomy, remains limited. This study aimed to comprehensively evaluate the electric field (E-field) induced in TMS operators under realistic working conditions, with a focus on how inter-subject anatomical variability affects compliance with international safety guidelines.

**Methods:**

Numerical simulations were performed using four anatomically detailed virtual human models exposed to a circular TMS coil in clinically relevant positions. Two coil heights (chest and abdomen) and three coil-to-operator distances (12 cm, 22 cm, 40 cm) were analyzed. The induced E-field was computed using magneto-quasi-static solvers, and the results were compared with the ICNIRP basic restriction for occupational exposure (1.13 V/m) and with the experimental threshold for peripheral neurostimulation (4 V/m). Whole-body percentiles, localized distributions, and anatomical cross-sections were evaluated to characterize inter-subject variability.

**Results:**

At 40 cm, all models and exposure scenarios were compliant with ICNIRP limits. At 22 cm, most configurations remained compliant, though some models slightly exceeded the reference level, particularly in the abdominal region. At 12 cm, the induced E-field systematically exceeded the occupational limit across all models, while remaining below the neurostimulation threshold. Anatomical features, such as subcutaneous adipose tissue thickness and tissue discontinuities, were found to strongly influence field localization and intensity, especially at closer distances.

**Conclusion:**

Operator exposure to TMS is influenced by both coil positioning and individual anatomical characteristics. While increasing distance ensures compliance and reduces variability, closer configurations may require tailored assessments or mitigation strategies. These findings highlight the importance of considering inter-subject variability in the evaluation of operator exposure and suggest that anatomical models may underestimate variability in real clinical contexts. A limitation of this study is the assumption of a static operator not manually holding the coil, a common condition in clinical practice that warrants further investigation.

## 1 Introduction

Transcranial magnetic stimulation (TMS) is a non-invasive neurostimulation technique that has gained considerable clinical and scientific relevance over the past two decades (1). Introduced in the early 1980s (2), TMS delivers pulsed magnetic fields to modulate neural activity and investigate brain function (3). Its use is expanding rapidly in both clinical practice and research, due to its ability to reach cortical areas without pain or surgical procedures (4). TMS has been approved for the treatment of various neuropsychiatric and neurological conditions, including major depression (5, 6) obsessive-compulsive behavior (7), and substance dependence (8) with regulatory support from the U. S. Food and Drug Administration (FDA) (9) and CE marking in Europe (10). It is also employed for neuromodulation and functional mapping in cases such as multiple sclerosis (11), Parkinson’s disease (12), motor neuron pathologies (13), and spinal cord injury (4). A major advantage is its improved tolerability compared to electrical stimulation, particularly in terms of reduced scalp pain for patients (4). A TMS system consists of a pulse generator and a stimulation coil. Among the most commonly used geometries are the circular coil and the figure-of-eight coil (14–20). The circular coil, composed of a single loop with multiple windings, produces a broad stimulation pattern, while the figure-of-eight coil provides more focal stimulation. Despite these differences, both coil types generate intense, time-varying magnetic fields in the range of 1–3 T (21, 22). These magnetic fields, in turn, induce electric fields (EFs) in neural tissues, up to 100 V/m, via Faraday’s law (23, 24), allowing controlled stimulation of specific brain regions.

During treatment sessions, clinicians often hold or support the coil in place above the patient’s scalp, positioning themselves near the source of the magnetic field. This operating condition leads to variable magnetic field exposure of the clinician’s body, which causes induced electric fields, which may vary depending on coil geometry, stimulation parameters, and positioning. Previous studies (25–28), based on numerical simulations, have reported that, under certain exposure conditions, the magnitude of the induced EF in the clinician can exceed the occupational limits recommended by the International Commission on Non-Ionizing Radiation Protection (29). The increasing use of TMS has prompted recent regulatory attention. Indeed, the Commission Implementing Regulation 2022/2347/EU (30) now classifies TMS as a Class III device under Regulation (EU) 2017/745 (31). However, unlike magnetic resonance imaging (MRI), for which technical standards such as EN 60601–2-33 (32, 33) provide support for exposure evaluation, TMS currently lacks harmonized protocols or standardized assessment methods, particularly for evaluating exposure of those operating with the equipment. An additional aspect that remains insufficiently addressed is how individual anatomical differences influence electromagnetic field (EMF) exposure. Parameters such as body composition, tissue distribution, and structural features can significantly affect the spatial pattern and intensity of the induced EF. While in other similar research topics, the role of inter-individual biological variability has been explored in terms of response sensitivity (34, 35) and thresholds (36–42), few studies (43, 44) have examined the influence of anatomical variation on EMF field distributions from a modeling standpoint. Computational human models, which vary by gender, age, and body size, are frequently used in EMF dosimetry. A recent study involving wearable antennas demonstrated significant differences in EF distribution across anatomies, with reported variations exceeding 100% depending on gender (45). However, application to modeling exposure conditions in TMS, particularly for operators, has not been widely investigated. Indeed, in TMS, studies of inter-subject variability have thus far focused almost exclusively on patient exposure (16, 46), where it has been shown that EF distribution can be strongly influenced by tissue interfaces and anatomical details.

The study here proposed addresses these gaps by introducing a comprehensive and structured numerical dosimetric analysis of EMF exposure in TMS operators using four anatomically realistic virtual human models. The models represent different body types and were exposed to a circular coil placed in two positions (at chest and abdominal level) and three distances (12 cm, 22 cm, and 40 cm), the first two based on scenarios commonly observed in clinical settings and the last one taken from a recommendation in Rossi et al. (47). These configurations are based on previous measurement campaigns (48) and literature-defined working conditions (26–28, 47, 49–51). The objective is to assess how variations in anatomy and tissue structure affect the distribution and magnitude of the induced EF in the operator, and to characterize how this dependence becomes more pronounced at closer proximities to the coil. In addition, the EF values obtained are examined in relation to current exposure limits, including the ICNIRP guidelines and the threshold level (52), cited in the context of potential peripheral nerve stimulation. It should be noted that in the European context, worker exposure to EMF is regulated by Directive 2013/35/EU (53), which establishes minimum health and safety requirements regarding the exposure of workers to electromagnetic fields. However, since the limit structure adopted by the Directive is conceptually derived from the ICNIRP guidance, this paper will refer to the latter for the assessment of worker exposure, given its international relevance.

This work highlights the importance of incorporating anatomical specificity into EMF exposure assessments and contributes to a more detailed understanding of how physical factors influence field distributions in practical applications.

## 2 Materials and methods

### 2.1 Numerical exposure scenarios

The exposure scenarios replicated in this study are based on the measurement campaign by Bogi et al. (54) where the commercial Magstim MAG-9784-00 circular coil fed by the BiStim appliance (55, 56) was experimentally characterized. The TMS feeding current signal can be assimilated to a pure sinusoid (50, 57) at the equivalent frequency of 1 kHz. All the features of the modelled TMS system are reported in Table 1.

**Table 1:** The characteristics of coils.

Parameters	Circular coil
	Magstim MAG-9784-00
Power system	*BiStim*
Frequency	1 kHz
Current (100% MSO*)	9.7 kA
Inner diameter	7 cm
Outer diameter	12.2 cm
Turns	14

*Maximum TMS simulator output.

It is noteworthy that, although figure-of-eight coils are most commonly used in clinical practice because of their higher focality, a circular coil was chosen in this study since it generates a broader magnetic field distribution, making it suitable for assessing the worst-case scenario in terms of operator exposure. This approach is consistent with previous numerical and experimental investigations [e.g., Karlström et al. (26); Bottauscio et al. (50); D’Agostino et al. (28)]. Furthermore, as already mentioned, the chosen coil model was experimentally characterized by Bogi et al. (54), ensuring a direct correspondence between the numerical simulations and the measured field data.

The TMS coil was modeled by including only the windings, omitting the external casing, in accordance with standard practices in TMS numerical modeling. In addition, the coil was approximated as a dimensionless wire, as commonly adopted in the literature for computational efficiency and fidelity to induced field behavior (58, 59). To characterize the model and validate its electromagnetic behavior, we conducted a comparison between simulation results and measurements obtained from a dedicated experimental campaign. Specifically, magnetic field intensities were recorded at various spatial locations around the actual coil when operated at 70% of the maximum stimulator output (MSO). The same configuration was implemented in the numerical platform Sim4Life (60) (v7.0, ZMT, Zurich MedTech AG). The comparison showed a strong agreement between simulated and measured magnetic field distributions when the simulated coil was driven by a peak current of 6.8 kA, corresponding to 70% MSO, thus confirming the model’s representativeness of realistic operating conditions (more details in Section 1 of Supplementary material). This current level was therefore used as the excitation input in all subsequent simulations, as it also reflects a typical setting in real TMS procedures.

Following model validation, we replicated a typical clinical working scenario in Sim4Life v7.0, including both the operator (clinician) and a simplified representation of the patient. For the patient, we used the SAM phantom (Standard Anthropomorphic Model) provided within Sim4Life, which is based on the specifications from IEEE Standards Coordinating Committee 34, Sub Committee 2, Working Group 1 SCC34/SC2/WG1. This phantom includes only two tissue layers: a skull shell and a brain-equivalent fluid, to which we assigned the respective low-frequency electrical properties (conductivity values) from the IT’IS LF database (61, 62). The TMS coil was placed approximately 1 cm above the phantom’s head, ensuring a realistic coupling between the coil and the simulated patient. The inclusion of the patient’s head model allowed us to recreate a clinically meaningful spatial configuration for operator exposure assessment. However, it is essential to recognize that the focus of this study is exclusively on the electromagnetic quantities induced in the clinician’s body; the field distribution inside the patient’s head is outside the scope of this work.

**Figure 1:**
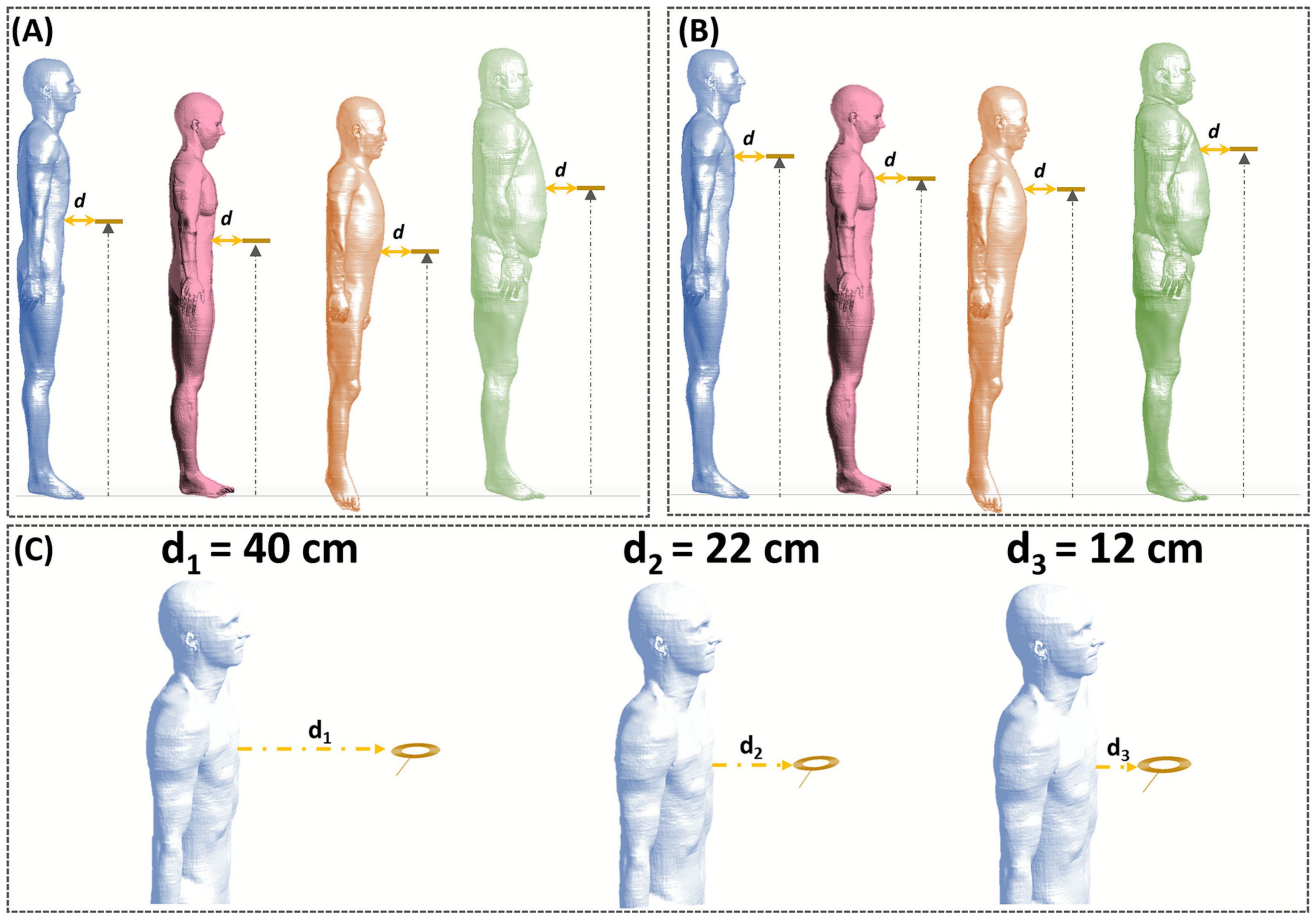
Coil position as a function of body size. The black arrow indicates the coil height; the yellow arrow indicates the distances *d*, measured at coil height and perpendicular to the body’s longitudinal axis. Panel **(A)** Abdominal and **(B)** chest exposure in the four human models: Duke (light blue), Ella (pink), Jeduk (orange), and Fats (green). Panel **(C)** Coil-to-body distance *d* used in the exposure scenarios here analyzed (40 cm, 22 cm, and 12 cm), illustrated for the chest case only.

Compared to our previous study (28), which analyzed two clinician orientations, four coil heights, and a single coil-to-operator distance of 12 cm, the present work focuses on a single operator orientation (sideways relative to the coil edge) and introduces a comprehensive analysis of three coil-to-body distances: 40 cm, 22 cm, and 12 cm (Figure 1C).

The expanded range of distances allows a more continuous investigation of how proximity influences operator exposure. Each distance was chosen based on methodological continuity, clinical realism, and regulatory relevance:

- Distance of 40 cm:

- Corresponds to the recommended minimum operator distance, in Rossi et al. (47).
- Is supported by experimental thresholds for ICNIRP Reference Levels (29): 40 cm (27), 70 cm (26), and 90 cm (49).
- Is supported by computational thresholds for compliance with ICNIRP Basic Restrictions (29): 38 cm (28), 64 cm (50), and 110 cm (49).

- Distance of 22 cm:

- Introduced as an intermediate and clinically plausible configuration, offering a practical compromise between close proximity and extended reach.
- Reflects a sustainable working posture for most clinicians, considering variability in arm length and handling technique.

- Distance of 12 cm:

- Used in our previous study (28), enabling direct comparison and methodological consistency.
- Represents a realistic clinical posture, where the operator’s arm is bent at ~90°, holding the coil close to the patient.
- Identified as a potential threshold for exceeding exposure limits in Rutherford et al. (61).

To investigate the impact of vertical positioning, we evaluated two coil heights, corresponding to anatomically relevant regions: the abdomen and the chest (Figures 1A,B).

The rationale for choosing these regions was twofold:

- The abdomen was previously identified as the worst-case exposure configuration (28);
- The chest was specifically included to evaluate scenarios involving the female human model, given its anatomical significance.

For all models, available in the Virtual Population (ViP v3.1) (62, 63), and imported into Sim4Life, the coil height was individually adjusted to match each clinician’s anthropometry, ensuring consistent targeting of the intended body region. Thus, for each anatomical model, the coil was positioned in such a way that its plane was approximately perpendicular to the body surface at the targeted anatomical level (chest or abdomen). The distances reported between the coil and the body (12, 22, 40 cm) indicate the minimum distance measured between the outer windings of the coil and the nearest point on the outer surface of the model (on the skin). In other words, the coil-to-body distance *d* consistently represents the shortest spacing between the coil windings and the body surface, measured at the height of the coil and perpendicular to the body’s longitudinal (head–to–feet) axis. The height of the coil was adjusted individually to fit the anthropometry of each model, ensuring consistent targeting between subjects.

Therefore, these operator-coil configurations were selected to reflect the postures most commonly adopted by TMS operators in clinical settings. Specifically, the distances of 12 cm and 22 cm reproduce scenarios in which the operator manually holds or supports the coil during patient stimulation, while the 40 cm distance corresponds to the minimum recommended safety spacing, as indicated by Rossi et al. (47) and other operator exposure studies. The associated coil heights (chest and abdomen levels) were aligned with the positions observed in real clinical sessions, as documented in D’Agostino et al. (28). Although the adopted postures do not explicitly include hand–coil interaction, it can be reasonably expected that the hands, being closer to the applicator, would experience a higher local magnetic field, which consequently induces a stronger E-field. Thus, this important aspect will be investigated in future work.

Figure 1 summarizes the simulation setup:

- Panels (A) and (B) show the four clinician models positioned sideways relative to the TMS coil at the two selected heights.
- Panel (C) presents the top view of the three evaluated distances: 40 cm, 22 cm, and 12 cm.

Across all conditions, dielectric properties of tissues were assigned using the IT’IS Low Frequency (LF) database v4.1 (62). Further details on the numerical implementation and hardware characteristics are reported in the Supplementary material.

The simulations were implemented with the Magneto-Quasi Static solver in Sim4Life software, which computes the Biot–Savart law as in Equation 1.


$$A(r)=\frac{\mu_{0}}{4\pi }\int_{\Omega }\frac{J_{0}(r^{\prime })}{|r-r^{\prime }|}d^{3}r^{\prime }$$ (1)

Where A is the magnetic vector potential, J_0_ is the current; μ_0_ is the magnetic permeability (constant over the entire domain Ω); *r* and *r*′ are the position vector inside the domain and the position vector of the source, respectively. Under the quasi-static approximation, the electric field *E* can be written as the Equation 2:


E=−jωA−∇V (2)

Where the E-field is composed of a primary field E_p_ ( −jωA ) and secondary field E_s_ ( −∇V ). The electric scalar potential V is computed by applying the finite element method (FEM) to Equation 3:


∇⋅σ∇V=−jω∇⋅(σA) (3)

Where *σ* is the conductivity of each tissue and *ω* is the angular frequency. This latter equation is valid in the hypothesis of ohmic current domination; hence, the contribution of the displacement is neglected.

Following ICNIRP 2010 Guidelines (29), all the human models were discretized using a uniform spatial resolution of 2 × 2 × 2 mm^3^ within the same simulation domain, resulting in a total cell count ranging from approximately 74 ÷ 110 MCells, depending on the specific human model. According to ICNIRP recommendations, the induced electric field must be evaluated as the vector average within contiguous 2 × 2 × 2 mm^3^ tissue volumes, and the 99th percentile of these averaged values should be used for comparison with the Basic Restrictions. Accordingly, in this study, the 99th percentile of the induced electric field distribution was adopted as the reference metric for comparison with the basic restriction, equal to 1.13 V/m (E_peak_) at the operating frequency of the analyzed coil.

## 3 Results

### 3.1 Assessment of operator exposure

To conduct the exposure assessment of the four subjects, compliance with the occupation limits for the three distances, and the two positions, was analyzed, considering a coil input current of 6.8 kA (70% MSO, as the real working condition). At this step, we performed a whole-body analysis of the induced E-field inside the human models, as suggested by the ICNIRP guidelines. Thus, in Reference source not found, we reported the 99th percentiles of the induced EF in the cases of exposure of the abdomen and the chest for the distance of 40 cm (Table 2) (more data for the three distances are reported in the Supplementary material). The values of the 99th percentile must be compared with the limit of 1.13 V/m (limit expressed as peak value at working frequency).

Considering this distance, the exposure scenario seems to be compliant with the ICNIRP limit, with all the 99^th^ percentiles of the induced E-field well below the limit (i.e., 1.13 V/m), with no relevant role of the body anatomy, tissues distribution and kind of exposure (abdomen or chest). These findings are in line with the *“expert guidelines”* (47) and with previous literature (26) confirming the importance of taking into account, as a method of reduction of the exposure, the distance from the source.

**Figure 2:**
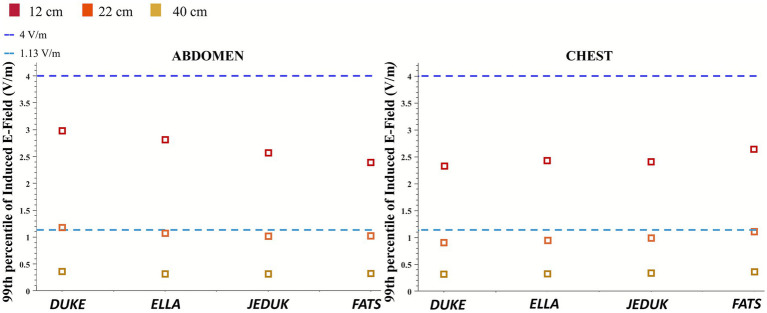
99th percentile of the induced E-field (70% MSO) for each body model, evaluated at three coil-to-body distances and for both exposure scenarios: coil positioned at abdomen height (left panel) and at chest height (right panel).

Figure 2 reports graphically the evaluated 99th percentile of the induced electric field produced in the operator’s body during TMS application, for both the evaluated exposure scenarios, with the coil at the height of the abdomen and at the height of the chest, and for all three coil-to-operator distances: 40 cm, 22 cm, and 12 cm. These distances reflect progressively closer operator positions, with 40 cm corresponding to the separation recommended in clinical safety guidance (47), while the shorter distances represent conditions possibly observed in routine practice. Two reference thresholds are indicated: the 1.13 V/m basic restriction set by ICNIRP 2010 for occupational exposure, and the 4 V/m level reported by So et al. (52), corresponding to the experimental threshold for peripheral neuronal stimulation in the low-frequency range. As already shown in Table 2, at a 40 cm distance, the induced E-field values remain consistently below the ICNIRP occupational limit. At 22 cm (data reported in Supplementary material), the 99th percentile values of the induced E-field increase modestly and approach or slightly exceed the 1.13 V/m limit in some scenarios, particularly at the abdominal level, indicating marginal compliance and it will be the object of further investigation in this paper. In contrast, at 12 cm (data reported in Supplementary material), the induced E-field systematically exceeds the ICNIRP suggested limit for both position cases and in all simulated conditions. The consistent exceedance of the occupational exposure limit at this close range highlights a clear possibility of overexposure if such proximity is maintained during TMS procedures, even though none of the assessed configurations reach the 4 V/m stimulation threshold. The purpose of Figure 2 is to show how inter-subject variability becomes increasingly relevant as the coil approaches the body. At the largest distance (40 cm), the 99th-percentile induced electric field values of the four models lie well below the exposure limit and minimal differences occur across subjects. As the coil-to-body distance decreases, a wider spread of the 99th-percentile values among models is observed, indicating that anatomical variability becomes more relevant at closer proximity.

**Table 2:** 99th percentiles of the Induced Electric Field (V/m) in the human models at 70% MSO for the distance of 40 cm in the two exposure scenarios.

Exposure scenarios		Duke	Ella	Jeduk	Fats
ABDOMEN	0.36	0.32	0.32	0.33
	CHEST	0.31	0.32	0.34	0.36

**Figure 3:**
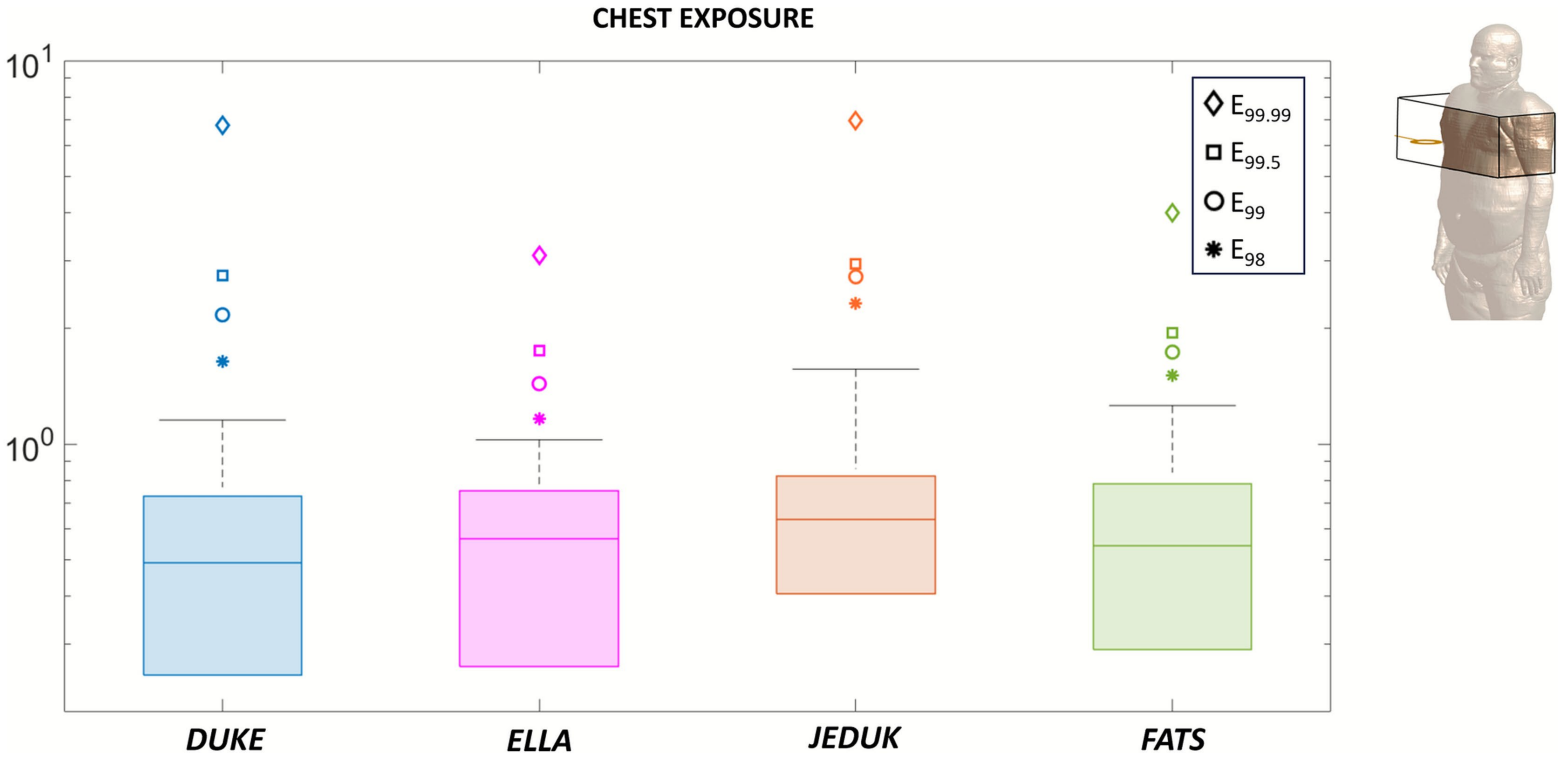
Exposure of the chest at 70% MSO. Box-plot of the E-field in SAT, fat, skin, and breast tissues, referred to a local area of the body in front of the coil at 22 cm. Upper whiskers identify 95th percentile. The outliers identified are the 98th, 99th, 99.5th, and 99.99th percentiles.

**Figure 4:**
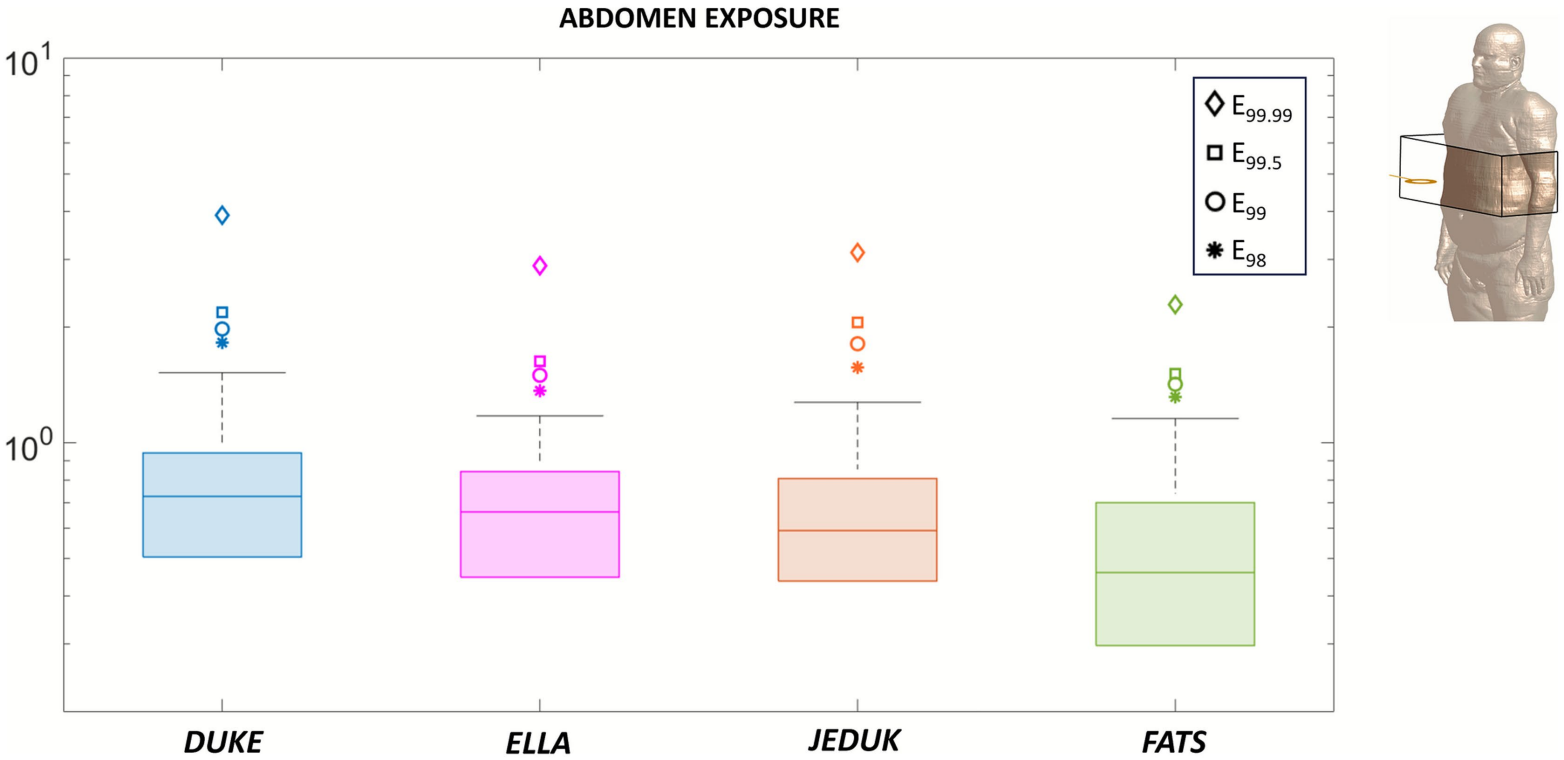
Exposure of the abdomen at 70% MSO. Box-plot of the E-field in SAT, fat, skin, and breast tissues, referred to a local area of the body in front of the coil at 22 cm. Upper whiskers identify 95th percentile. The outliers identified are the 98th, 99th, 99.5th and 99.99th percentiles.

To better understand the spatial characteristics of operator exposure in proximity to the TMS coil, we performed a localized analysis by restricting the evaluation of the induced electric field to a 24 cm-sided cubic volume centered at the coil height and positioned in the chest or in the abdomen regions, directly in front of the coil. This analysis was conducted for a coil-to-operator distance of 22 cm and applied to all four anatomical models, Duke, Ella, Jeduk, and Fats, as shown in Figures 3, 4 (see Table 3).

**Table 3:** Characteristics of the human anatomical models.

Human models	Age	Weight (kg)	Height (m)	Body structures	BMI (kg/m^2^)
Duke	34	70.2	1.77	319	22.4
Ella	26	57.3	1.63	312	21.6
Jeduk	33	64.5	1.62	1186*	24.6
Fats	37	119	1.82	247	36

*Nerves, muscles, veins, and arteries are separated for a neuronal analysis.

Figure 3 illustrates, through boxplot graphics, the distribution of the induced E-field within this localized region for chest exposure. The logarithmic scale well captures its full dynamic range. The upper whiskers are set to include up to the 95th percentile of the distribution, and the sole 98th, 99th, 99.5th and 99.99th percentiles are visualized as outliers. The median E-field values lie between approximately 0.4 and 0.9 V/m across all models, with Duke and Ella showing slightly lower medians compared to Jeduk and Fats. Despite the overall similarity in central tendency, noticeable differences emerge in the spread and upper whisker. Jeduk, in particular, presents a broader interquartile range. All models exhibit a regular distribution up to the 99.5th percentile, as testified by the relative proximities of these percentiles with the upper whisker of the distribution. Such outliers also represent localized peaks of induced fields likely associated with anatomical inhomogeneities. These results confirm that, even when focusing on a fixed anatomical region and under identical exposure conditions, inter-subject variability remains evident. Variations in body shape, tissue composition (e.g., fat vs. muscle), and internal organ geometry contribute to differences in local field distribution. While the median values remain below the ICNIRP occupational limit of 1.13 V/m in all cases, the presence of higher percentile and outlier values reinforces the importance of considering subject-specific anatomy when assessing occupational exposure.

The same procedure was followed for abdominal exposure, and the results are shown in Figure 4. Similarly, the distribution remains regular up to the 95th percentile. Across all subjects, the induced fields are confined below the occupational limit. More appreciable differences appear in the upper whiskers, which extend to slightly higher values in some models. Upper outliers reach lower values than those of chest exposure and remain confined within 4 V/m in all subjects. Interesting, Fats model shows the lowest median E-field among all subjects, and this downward tendency is consistent with the presence of a thicker adipose layer, whose lower electrical conductivity compared with other tissues, as muscle, tends to attenuate the induced E-field. Furthermore, a smaller interquartile range can be observed, showing a reduced spread of the induced values.

This localized approach offers a more targeted perspective on exposure conditions and highlights the relevance of individualized modeling in safety assessments near the TMS coil.

**Figure 5:**
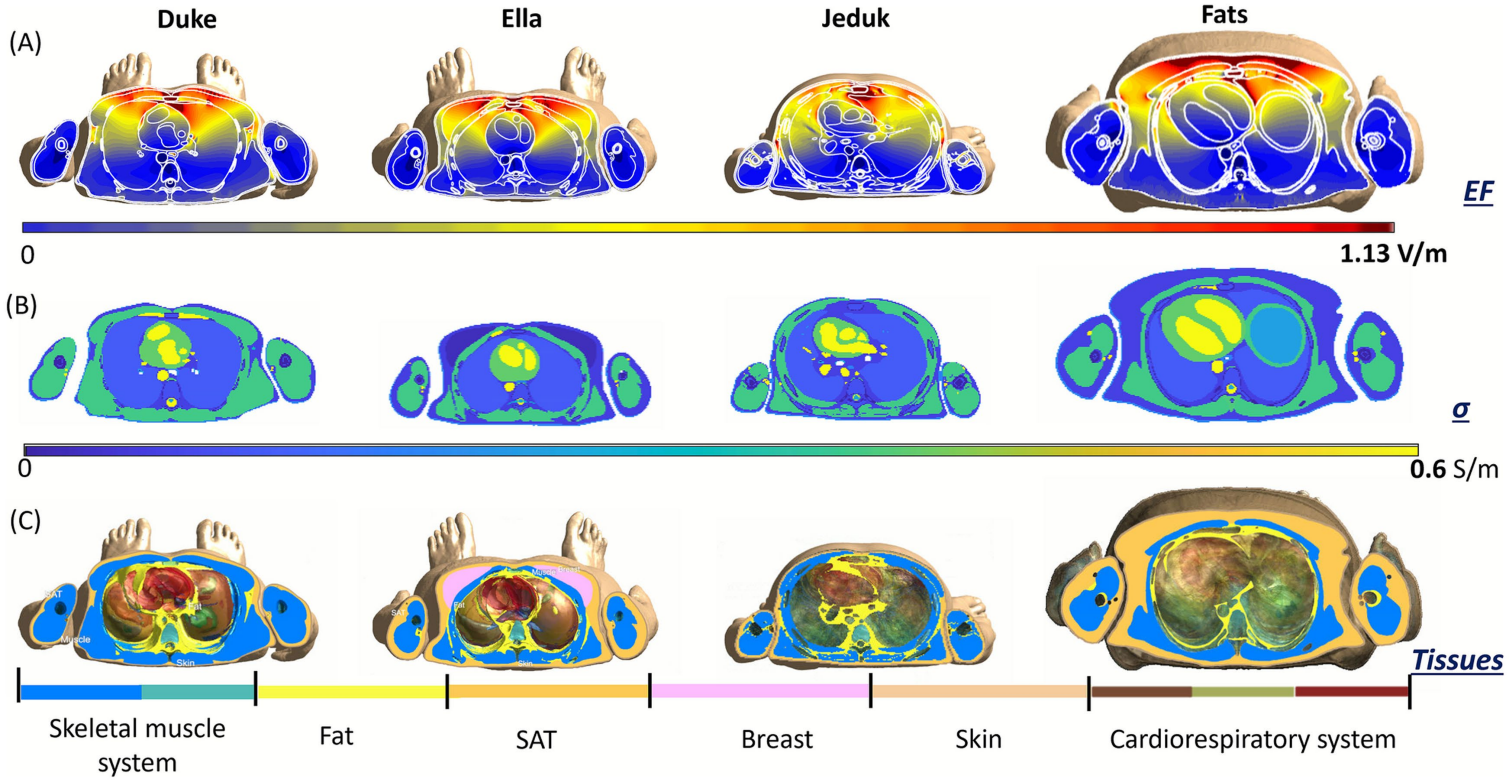
Transverse slice, coil at the height of the chest at 70% MSO: induced E-field **(A)**, the conductivity of tissues **(B)**, and body tissues **(C)**. Human models at 22 cm distance from the coil.

**Figure 6:**
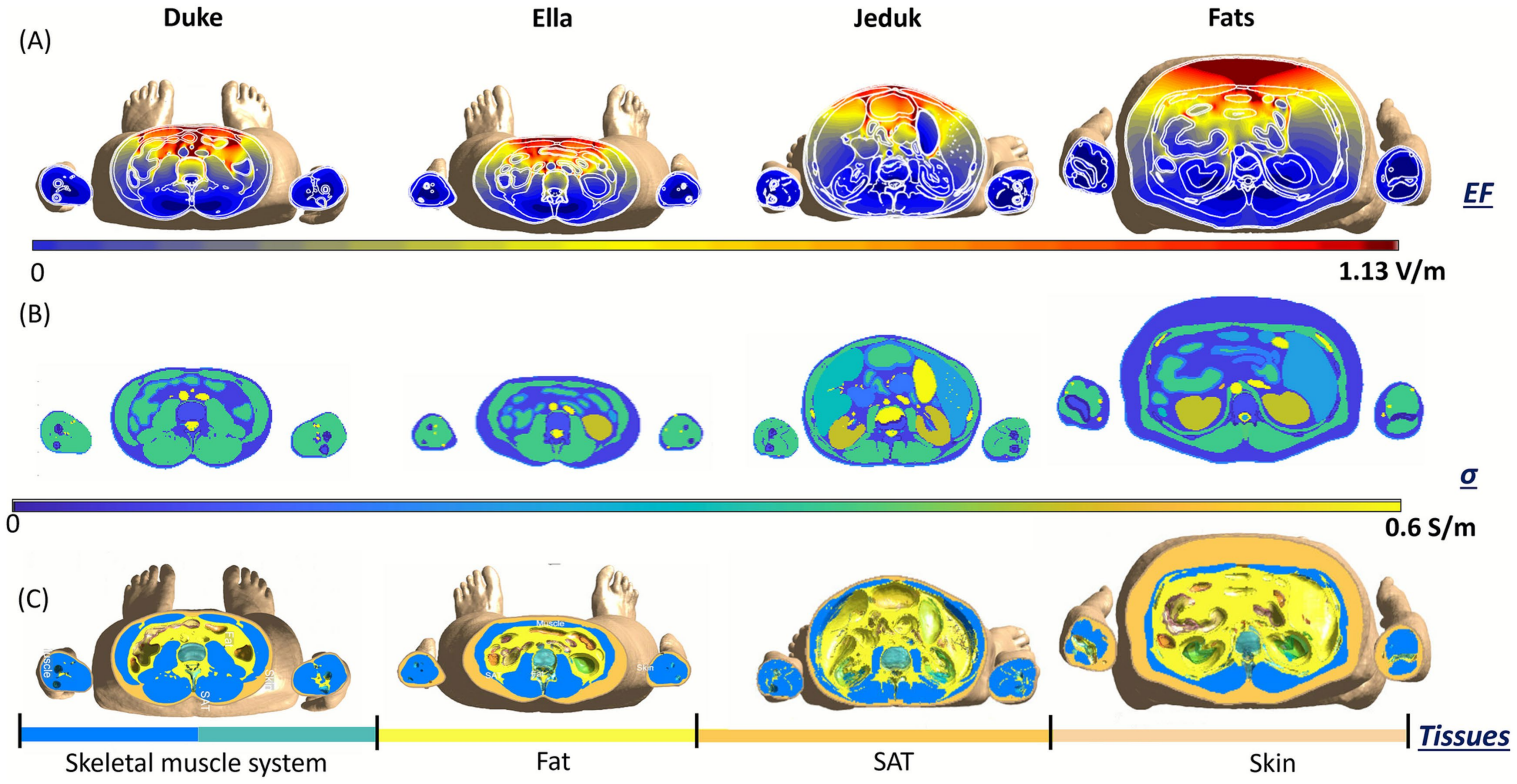
Transverse slice, coil at the height of the abdomen at 70% MSO. Induced E-field **(A)**, conductivity of tissues **(B)**, and body tissues **(C)**. Human models at 22 cm distance from the coil.

To further explore the anatomical determinants of inter-subject variability in induced electric fields, we examined transverse cross-sections taken at the same coil height in two anatomical regions: the chest and the abdomen. Figures 5, 6 present a comparative view across the four anatomical models, Duke, Ella, Jeduk, and Fats, providing three aligned data layers for each region: the spatial distribution of the induced E-field (top row), the local tissue conductivity (middle row), and the anatomical segmentation of relevant tissue groups (bottom row). In the chest region the top row (Figure 5A), reveals that, despite identical coil-to-body configurations, the magnitude and spatial pattern of the E-field vary notably among models. Duke and Jeduk show more concentrated and intense fields, with maxima located in the upper thoracic region and extending laterally. Fats, in contrast, presents a more diffuse field distribution, consistent with the presence of a thick subcutaneous adipose tissue (SAT) layer that likely dampens field penetration. Ella exhibits a symmetric and relatively broad field distribution, but with generally lower amplitude. The second row (Figure 5B) shows the corresponding tissue conductivity maps, highlighting significant differences in both distribution and magnitude of conductive structures. In Duke and Jeduk, high-conductivity values (approaching 0.6 S/m) align with thoracic muscles and central organs. Fats shows a predominance of low-conductivity tissues (fat and SAT). Ella displays a more centralized region of moderate conductivity, aligned with breast and upper thoracic tissues. The third row (Figure 5C), showing anatomical segmentation, helps interpret these patterns. Fats presents a large volume of SAT and skin, and relatively little directly exposed muscle tissue, which contributes to spread-out E-field distribution. Duke and Jeduk display more prominent skeletal muscle and cardiorespiratory structures near the surface, corresponding to more localized E-field enhancement. Ella includes a distinct proportion of breast tissue, which may explain the observed symmetry and intermediate field characteristics.

In Figure 6, a similar comparison was conducted at the abdominal level. Again, the top row (Figure 6A), reveals marked differences in E-field magnitude and spatial localization. Jeduk and Duke show higher peak fields in deep abdominal tissues, particularly near the spine and major vascular structures, while Fats shows a pronounced attenuation of the field, with peak values remaining superficial and broadly distributed. Ella presents a smoother distribution with moderate intensity, reflecting a relatively homogeneous tissue structure. In the second row (Figure 6B), conductivity maps show that Jeduk and Duke include deeper regions of high conductivity associated with visceral organs and musculature. Ella presents smaller, more central conductive regions, while Fats is again dominated by low-conductivity SAT and fat, which results in both reduced E-field penetration and intensity. The anatomical segmentations in the third row (Figure 6C) provide further insight. Fats again displays a thick SAT envelope surrounding the torso, limiting the exposure of high-conductivity internal structures. Duke and Jeduk reveal substantial regions of muscle and internal organs within the field path, leading to more focused and intense E-field distributions. Ella shows a balanced configuration with a central arrangement of conductive organs and moderate SAT thickness, contributing to a field profile that is intermediate in both strength and spatial spread.

Taken together, these cross-sectional views provide a compelling illustration of how anatomical and dielectric properties modulate local E-field distributions in a subject-specific manner. In both chest and abdomen, models with greater superficial fat layers (e.g., Fats) show broader and weaker fields, due to the low conductivity of adipose tissues and the increased distance to deeper conductive structures. In contrast, models like Duke and Jeduk, characterized by thinner SAT and more prominent muscular and visceral anatomy, exhibit more localized and intense field peaks in areas of high conductivity. Ella, with more symmetric and anatomically balanced tissue distribution, consistently shows moderate field magnitudes and smoother patterns across both regions. These results highlight the importance of tissue composition and spatial arrangement, not just overall body size, in shaping local exposure levels. Notably, even when exposure conditions are geometrically identical, inter-subject anatomical variability significantly influences both the amplitude and spatial concentration of the induced electric fields. This detailed anatomical correlation confirms that subject-specific modeling is essential for accurately assessing occupational exposure in the context of TMS, especially when evaluating compliance with safety thresholds or optimizing operator positioning.

To deepen the understanding of coupling mechanisms between the TMS-induced electric field and human morphology, particularly at close proximity where inter-subject variability is most evident, we conducted a detailed analysis of exposure at a coil-to-operator distance of 12 cm. Although such a configuration is generally discouraged in clinical settings, it may still occur in some circumstances and thus warrants careful investigation. This analysis complements the results shown in Figure 2, confirming that at this short distance the induced fields frequently exceed the ICNIRP occupational limits, while remaining generally below the PNS stimulation threshold of 4 V/m.

**Figure 7:**
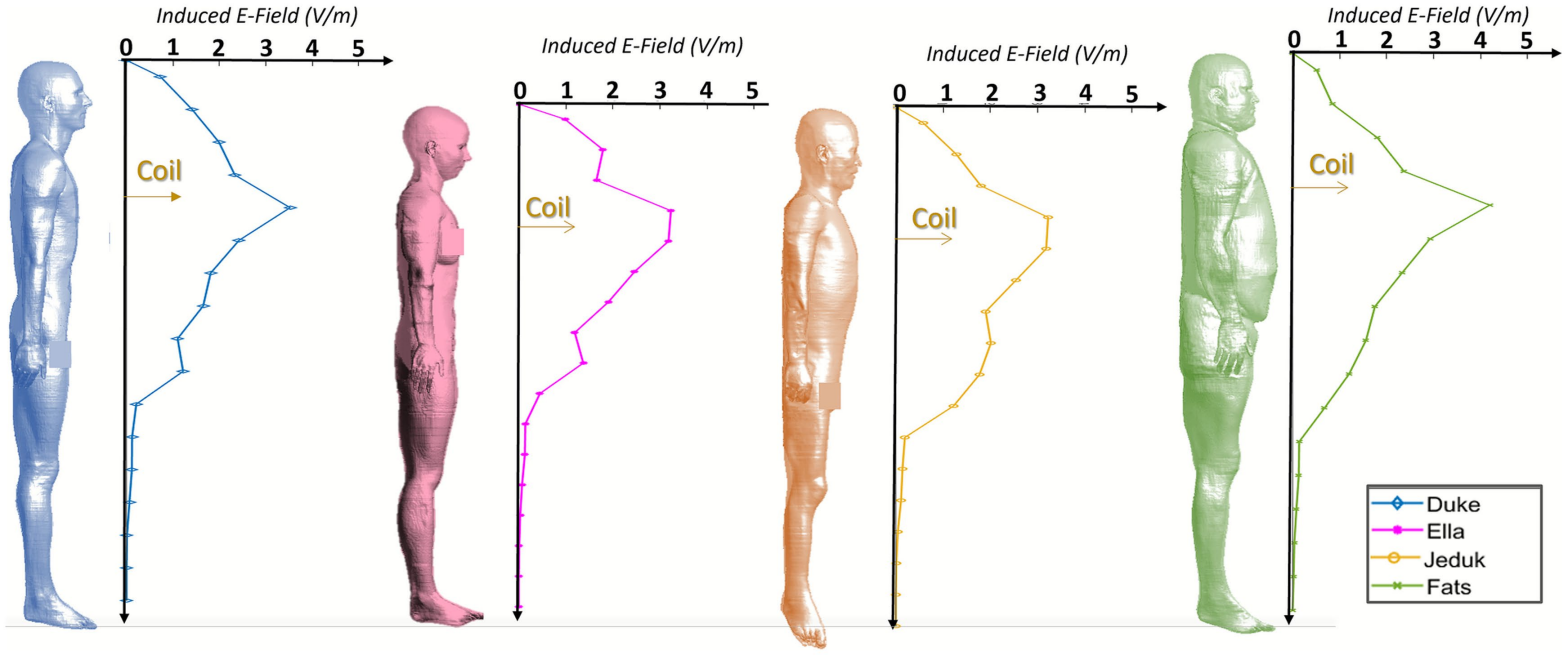
Exposure of the chest at 70% MSO. Induced E-field (99th percentile) distribution along the body of human models at 12 cm distance.

**Figure 8:**
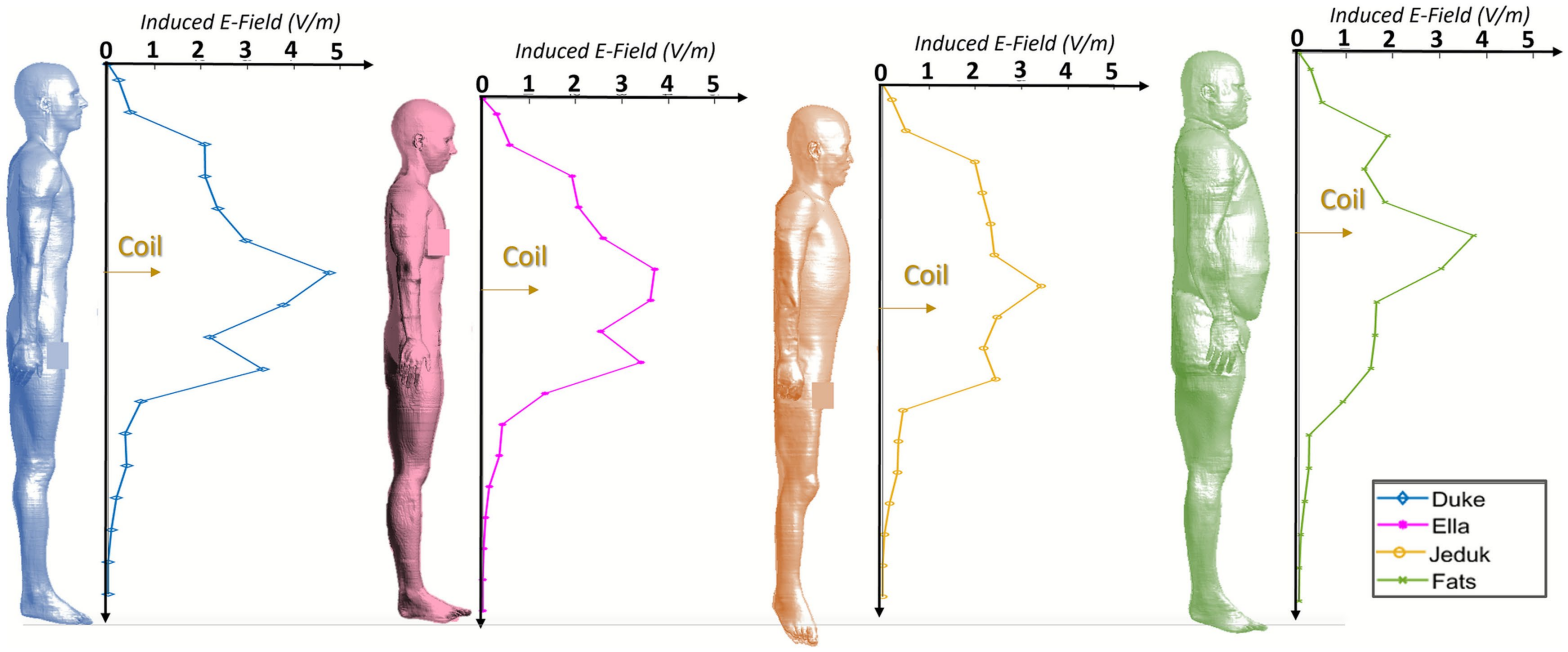
Exposure of the abdomen at 70% MSO. Induced E-field (99th percentile) distribution along the body of human models at 12 cm distance.

Each human model was divided into horizontal sections of 10 cm, from head to feet (for more details see Supplementary material). Within each section, the 99th percentile of the induced electric field at 70% MSO was evaluated. The resulting field trends along the body axis are reported in Figure 7 for chest exposure and in Figure 8 for abdomen exposure. Figure 7 shows that when the coil is placed at chest height, all models exhibit a peak induced field within the segment aligned with the source, with values ranging from approximately 2.5 to 3.7 V/m. In some models, such as Ella and Jeduk, the elevated field extends across multiple adjacent regions, suggesting that anatomical features like breast tissue or thoracic geometry may influence lateral field spread. The induced field consistently exceeds the ICNIRP occupational limit across multiple segments, although values remain below or close to, but not beyond, the 4 V/m threshold. A secondary peak in the lower torso or pubic area appears in most models, with a reduced intensity compared to the main maximum. In Fats, the field distribution is smoother and broader, with lower values, reflecting the role of thicker subcutaneous adipose tissue in modulating field penetration. Figure 8 presents the same analysis for the case in which the coil is positioned at abdomen height. Here, the maximum induced field reaches up to 4.75 V/m in Duke and around 3.7 V/m in Ella and Fats. These maxima are typically centered around the abdomen but may be accompanied by a secondary elevation in the lower pelvis. In all models, the ICNIRP occupational limit is exceeded over a considerable length of the torso, extending from the chest to the lower abdomen in Duke and Ella. In Jeduk, the induced field remains relatively constant around 2.5 V/m across several regions. In Fats, values gradually decrease from the abdomen to the feet without showing distinct secondary peaks, likely due to the thick adipose layer dampening field variations.

Overall, these results confirm that at 12 cm, the ICNIRP limit is exceeded in extended regions of the operator’s body, particularly around the coil height. While values approaching or slightly exceeding 4 V/m are observed in specific subjects and anatomical configurations, especially near the abdomen, these instances remain localized and do not suggest widespread neural stimulation risk. The data further highlights how individual anatomy, including tissue composition and body geometry, affects both the magnitude and spatial distribution of exposure. While close proximity should be avoided whenever possible to ensure compliance and reduce variability, the findings suggest that even under these less favorable conditions, peripheral neuronal stimulation thresholds are generally not exceeded.

## 4 Discussion

This study presents a comprehensive numerical evaluation of operator exposure to TMS, with a particular focus on how anatomical variability affects the spatial distribution and intensity of the induced electric field in the operator’s body. Extending prior work that considered a single anatomical model and a limited number of exposure configurations, the present analysis introduces an inter-subject comparison involving four anatomically diverse virtual human models exposed to a circular TMS coil at varying heights and distances. This expanded dataset, developed through a comprehensive approach, allows for a more exhaustive and representative understanding of how individual physical characteristics influence compliance with international exposure limits. It is worth noting that the observed inter-subject variability can provide useful insight to refine exposure assessment and improve control within the plausible range of operator exposure, rather than indicating specific individual sensitivity. From a regulatory standpoint, the same measures should apply to all operators. Considering human variability helps in defining appropriate approaches, including conservative evaluation of worst-case conditions, technical solutions (53) to limit exposure, and organizational measures such as training and task planning. Adjustment of the stimulator output, when feasible, can also contribute to maintaining exposure at prudent levels.

The first observation concerns the overall impact of proximity to the source on exposure. At a coil-to-body distance of 40 cm, all configurations remained within the ICNIRP occupational basic restriction of 1.13 V/m, regardless of coil height or anatomical model. This supports existing recommendations indicating that such distance provides a robust safety margin and minimizes the influence of anatomical variability (47). Indeed, at this distance, exposure levels are consistently low across chest and abdominal configurations, with minimal inter-subject variability, indicating that anatomical features and tissue composition have a negligible influence, and confirming the effectiveness of this separation as a protective measure (Table 2).

At an intermediate distance of 22 cm, the exposure values increase, and the role of anatomical features becomes more evident. While some models remained compliant, others, particularly when exposed at the abdominal level, slightly exceeded the ICNIRP suggested limit. This condition represents a transitional zone where exposure outcomes depend strongly on both coil height and individual anatomy. Notably, the analyses conducted at this distance provided valuable insight into the role of individual tissues: differences in composition and geometry begin to affect both the magnitude and spatial distribution of the induced field, even though values remain below neurostimulation thresholds (Figures 5, 6).

At the shortest distance of 12 cm, exposure levels are consistently above the ICNIRP occupational limit for all configurations. The 99th percentile of the induced E-field in these cases exceeded 1.13 V/m in all models and in both anatomical regions. However, the field intensities remained below or only approached the 4 V/m threshold for peripheral neural stimulation (52), indicating that while regulatory limits may be exceeded, the risk of acute neurophysiological effects remains low under typical operating conditions. Importantly, as with the 22 cm case, and even more so due to the close proximity between the coil and the body, this distance provided a valuable opportunity to analyze the coupling mechanisms between the EMF source and anatomical structures and elucidate the role of anatomical features, such as subcutaneous adipose tissue thickness and tissue discontinuities, in field localization and intensity. Still, the data confirms that this proximity should be avoided whenever possible, as it leads to the highest degree of inter-subject variability and the most extensive regions above ICNIRP limits exposure.

A central result of the study is the clear influence of individual anatomy on local E-field distribution. Differences in subcutaneous fat thickness, muscle mass, and tissue interfaces play a crucial role in shaping exposure. This is particularly evident at 12 cm, where the contrast between anatomies such as Fats (with thick subcutaneous adipose tissue) and Jeduk (with relatively low adipose coverage and high tissue discontinuity) leads to substantial differences in E-field magnitude and spatial concentration. The longitudinal profiles of the 99th percentile along the body axis (Figures 7, 8) demonstrate how anatomical structure determines not only the amplitude of the peak field but also its location and spatial extent.

Localized cross-sectional analyses at both chest and abdomen levels (Figures 3–6) reinforce these findings. In the chest, symmetric or homogeneous tissue arrangements, such as those in Ella, result in more uniform field distributions, while subjects with abrupt conductivity changes, such as Duke and Jeduk, show more focused and intense peaks. In Fats, the presence of thick adipose tissue leads to a broader, lower-magnitude field spread, consistent with its low conductivity. Similar patterns are observed in the abdomen, with the added effect of deeper organ structures influencing the spatial localization of peak exposure.

From a practical standpoint, these findings underline the importance of individualized exposure assessments, particularly when procedures involve closer-than-recommended coil placements. While 40 cm offers a conservative and generally safe configuration, exposure at 22 cm seems still acceptable in most cases, though subject-specific anatomical features should be considered. At 12 cm, the combination of increased field strength and variability suggests that careful control of both positioning and stimulation intensity is necessary to remain within appropriate operating conditions.

The results also support the potential role of adjusting stimulator output as a complement to spatial measures. For example, reducing the %MSO could compensate for short coil-to-body distances in specific individuals. However, given the variability across anatomical models, such adjustments should be informed by simulation or empirical assessment, rather than applied uniformly. Furthermore, it is relevant to mention that the numerical results are subject to uncertainty (64). Reported uncertainties in computational dosimetry are typically on the order of tens of percent or more, depending on the methodological used for the analysis (65) (e.g., coil modeling, voxel discretization, variability in dielectric properties, and posture assumptions); however, the use of the 99th percentile helps to reduce the staircase (66–68). Given these uncertainties, exposure conditions with induced fields close to the basic restriction should be interpreted conservatively. Therefore, mitigation measures (e.g., increased distance or lower stimulator output) become particularly important.

In conclusion, this work emphasizes that both coil distance and operator anatomy significantly affect the exposure to TMS. While greater distances reduce inter-subject differences and ensure compliance with regulatory limits, close proximity introduces more complex dependencies on tissue structure and distribution. These findings suggest that guidelines and clinical practices should consider not only general spatial recommendations but also the potential influence of individual variability, particularly in scenarios where maintaining distance is not always feasible.

## 5 Conclusion

This study provides a detailed numerical assessment of operator exposure to TMS, with a focus on how anatomical variability among clinicians influences the distribution and intensity of the induced electric field. By simulating a range of realistic exposure conditions involving different coil-to-body distances and anatomical regions, and by comparing four distinct virtual human models, we demonstrate that individual physical characteristics can significantly affect local exposure levels, particularly at short distances from the coil.

The results confirm that a coil-to-operator distance of 40 cm ensures compliance with ICNIRP basic restrictions across all models and configurations, largely mitigating the influence of anatomical differences. At 22 cm, exposure conditions lie near the boundary of compliance, with some models slightly exceeding the limit depending on coil height and tissue composition. At the shortest tested distance, 12 cm, the induced electric field systematically exceeds the occupational limit in all models, although values generally remain below or close to the experimental threshold for peripheral neural stimulation. These findings reinforce that distance is a key determinant of exposure and variability, but also that anatomical features such as subcutaneous fat distribution, tissue discontinuity, and internal geometry can substantially influence the spatial profile and magnitude of the induced field. As such, assessments based on a single reference model may not fully capture the range of possible exposure outcomes in clinical settings, particularly when close proximity to the coil is required.

While this study considered representative and well-characterized exposure conditions, it also has certain limitations. In particular, the operator models were positioned in static postures and did not manually hold the coil, as often occurs in real clinical practice despite the availability of robotic or mechanical support arms. The influence of arm position, hand contact, and local field enhancements near the upper limbs was therefore not evaluated and should be addressed in future investigations to further improve the validity of the assessment. In addition, as with any numerical dosimetry study, the results carry an intrinsic level of computational uncertainty linked to model discretization, material properties and solver settings. In this work, we adopted modelling choices that have been validated in the literature and are known to keep such uncertainty within acceptable bounds. A full uncertainty-propagation analysis lies outside the scope of the present study and will be considered in future developments.

Overall, these results highlight the importance of incorporating inter-subject variability into evaluations of operator exposure for TMS. They support current distance-based recommendations but also suggest that a more individualized or conservative approach may be warranted when coil proximity cannot be avoided. The numerical framework developed here may serve as a reference for refining exposure assessments and guiding the development of practical safety guidelines that better account for the diversity of clinical operators.

## Data Availability

The original contributions presented in the study are included in the article/Supplementary material, further inquiries can be directed to the corresponding author.
