# Supplementary material for: Intersubject variability in a comprehensive numerical assessment of operator electromagnetic exposure to TMS
Source: Front Public Health. 2026 Feb 2;13:1705893. doi: 10.3389/fpubh.2025.1705893 (PMC12908317; doi:10.3389/fpubh.2025.1705893)
Supplement: Supplementary file 1 [file Supplementary_file_1.pdf]

Supplementary Material

Intersubject variability in a comprehensive numerical assessment of operator electromagnetic exposure to TMS

S. D'Agostino[1], M. Colella[1], R. Falsaperla[2], M. Liberti[1], F. Apollonio[1,*]

[1]Department of Information Engineering, Electronics and Telecommunications, Sapienza University of Rome, Italy.

[2]Department of Occupational and Environmental Medicine, Epidemiology and Hygiene, Italian National Institute for Insurance against Accidents at Work, Rome, Italy.

*** Correspondence:**
Francesca Apollonio
francesca.apollonio@uniroma1.it

1 Characterization and validation of the circular coil

The device chosen for the dosimetric analysis has been derived from experimental data acquired during a measurement campaign conducted by the group of INAIL Research Center in November 2009 and is available on the Physical Agents Portal (1). During this campaign, the commercial Magstim MAG-9784-00 circular coil fed by the BiStim appliance(2–4) has been used. In Table 1, the features of the entire system are reported:

Table 1- Features of the devices

	**Circular coil Magstim MAG-9784-00**
**Power system**	BiStim
**Frequency**	1 kHz
**Current (MSO*)**	9.7 kA
**Inner diameter**	7 cm
**Outer diameter**	12.2 cm

*MSO: maximum stimulator output

The current signal that flows inside the TMS is assimilated to a pure sinusoid at equivalent frequencies obtained from the pulse period, as literature studies have shown that this assumption leads to a negligible error (5,6) when compared with the time-dependent signals. The experimental acquisitions have been conducted in a medical center and the measurement conditions have been provided considering the machine working in real operating conditions (70% MSO). The presence of the patient's head was reproduced by a phantom consisting in a plastic container (3 L capacity) filled with a 0.01 molar solution of NaCl in water, placed as in Supplementary Figure 1. The circular coil was

placed in contact with the container and oriented with axis parallel to the ground. The measuring points were chosen both at the position normally occupied by the operator and along the radial and axial direction of the coil to reconstruct the spatial distribution of the field. It is declared that the estimated uncertainty about the measures is 5%. In Supplementary Figure 1, is reported the measurement setup.

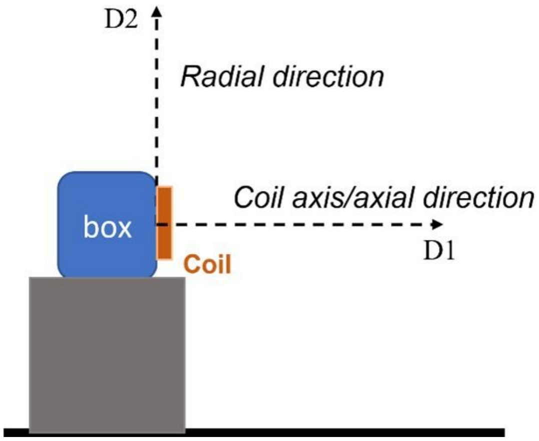

Figure 1. Measurement setup

We define D1, as the axial direction, whereas D2 is the radial direction. The measurements of the magnetic flux density (B-field) recorded during the campaign are reported in Table 2:

Table 2- Measured B-Field

	**POINTS OF MEASURE**		
	D1 [cm]	D2 [cm]	B(μT)$_{peak}$
**1**	4	0	667000
**2**	64	0	494
**3**	96	0	153
**4**	0	32	1827
**5**	0	64	251

The second step is to reproduce and numerically model the source validating its performances through a comparison with the values obtained during the experimental campaign. Therefore, following the features reported in Table 1, the coil model was reproduced using the numerical software Sim4Life (v.7, ZMT, Zurich MedTech AG). In the environment of simulation, it consisted only of the windings, without the outer coating, as done by current literature on TMS modeling(7). Therefore the coil was reproduced in the simulation environment as dimensionless wires placed at the center of the real wires(7,8) This implied a 2D coil approximation that may slightly underestimate the induced E-field, but the overall error was typically below 2%(7,9) in the range of work frequency here considered. In Supplementary Figure 2, it is reported the modeled coil in numerical software and the distribution of the magnetic field produced.

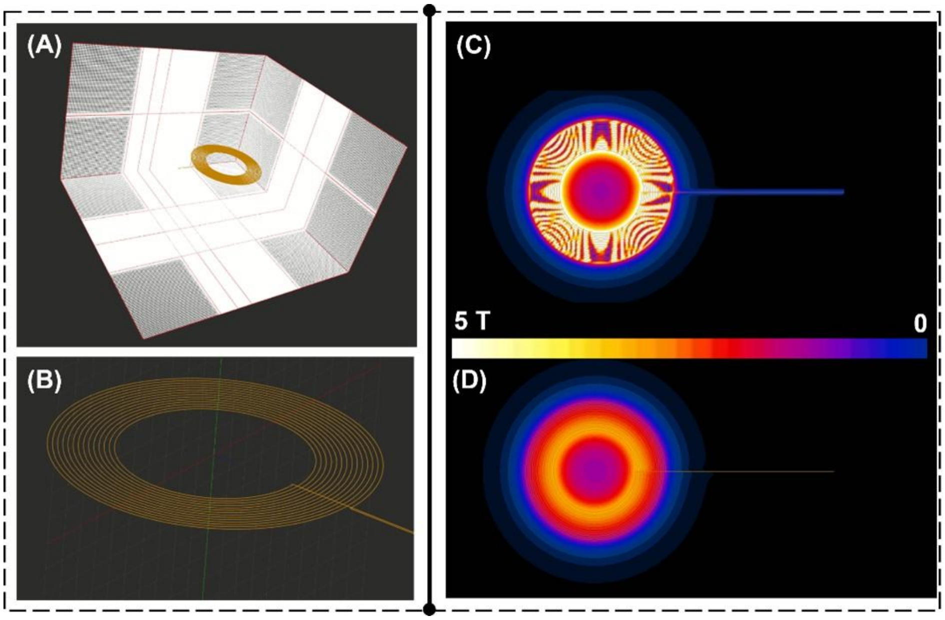

Supplementary Figure 2. Modeled coil in simulation environment: (A) coil in the grid (mesh) set in the simulation environment, (B) circular coil numerical model, (C) distribution of the magnetic flux density B(T) on the surface of the coil in corresponding of the windings, (D) distribution of B(T) in corresponding on the external surface in the position of the coating.

At this point, the analysis of the B-field around the coil is detected. Since we have the exact position in which the B-field is recorded, we move in the same direction in the simulation environment to compare the B-field. Supplementary Figure 3 shows the behavior of the B-field, with the maximum peak achieved in the corresponding of the inner turn of the coil (as the geometry of the coil suggests).

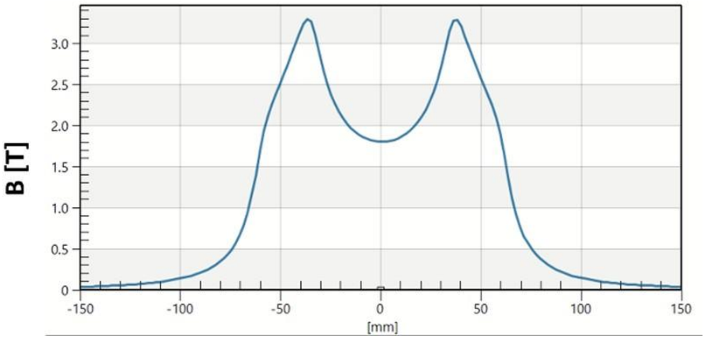

Supplementary Figure 3: B-field (T) along the line through the center of the coil in the outer surface.

The comparison with experimental measurements, along the two directions, is summarized in Tables 3 and 4:

Table 3 - Comparison of measured and simulated B (mT) along the radial direction

Distance	**Measured values** 70% MSO	**Dosimetric data** 70% MSO
32 cm	1.827	2.2
64 cm	0.251	0.268

Table 4 - Comparison of measured and simulated B (mT) along the axial direction

Distance	**Measured values** 70% MSO	**Dosimetric data** 70% MSO
4 cm	667	669.9
64 cm	0.494	0.52
96 cm	0.153	0.158

Taking into account the measurement error declared, we can conclude that the source modeled in the numerical environment returns roughly the same values recorded during the measurement campaign, with a maximum percentage variation, between the measured and simulated value, of 5.26%. Thus the coil model works properly as the real one.

2 Induced Electric Field in the four models for the three distances and two anatomical districts

Table 5. Induced Electric Field (V/m) in the human models at 70% MSO for the three distances and the two body districts

			DUKE	ELLA	JEDUK	FATS
40 cm	ABDOMEN	99th	0.36	0.32	0.32	0.33
		99.9^{th}	0.63	0.48	0.52	0.45
	CHEST	99th	0.31	0.32	0.34	0.36
		99.9^{th}	0.57	0.50	0.69	0.55
22 cm	ABDOMEN	99th	**1.18**	1.07	1.02	1.02
		99.9^{th}	1.99	1.66	1.63	1.44
	CHEST	99th	0.90	0.95	0.99	1.11
		99.9^{th}	1.61	1.46	2.16	1.70
12 cm	ABDOMEN	99th	**2.98**	**2.82**	**2.57**	**2.39**
		99.9^{th}	5.12	4.37	4.06	3.88
	CHEST	99th	**2.33**	**2.43**	**2.40**	**2.63**
		99.9^{th}	4.15	3.86	5.68	4.39

Comparison with established stimulation thresholds (10) shows that localized field peaks observed at 12 cm approach magnitudes relevant for peripheral nerve activation, yet remain far below values associated with cardiac stimulation, since the literature indicates that the thresholds for direct cardiac excitation range between 6.2 - 12 V/m (11–15). However, the evaluation of the E-field within the cardiac tissue is conducted to provide the basis for a possible future study that could include detailed anatomy of the heart and other parts of the body, such as the hand, in order to refine these evaluations. Table 6 reports the percentiles evaluated in the heart tissue for the case of the exposure of the chest, which directly involves the district of the heart.

Table 6. Percentiles of induced E-field (V/m) in heart tissues

Exposure *70% MSO*		DUKE	ELLA	JEDUK	FATS
**HEART**	*99th*	2.08	1.84	2.04	1.77
	99.9th	2.51	2.16	2.46	2.09

3 Exposure to circular coil: local peak in the transverse slice of the body

Referring to the Results paragraph of the main text, are here reported the figures showing the local peak found in the abdomen and chest exposure respectively, for the distance equal to 12 cm.
First, the peak values found in the case of abdomen exposure are highlighted in light green, Supplementary Figure 4. The body slices in the transversal plane, at the height of the abdomen, precisely corresponding at the position of the coil, are reported for each model.

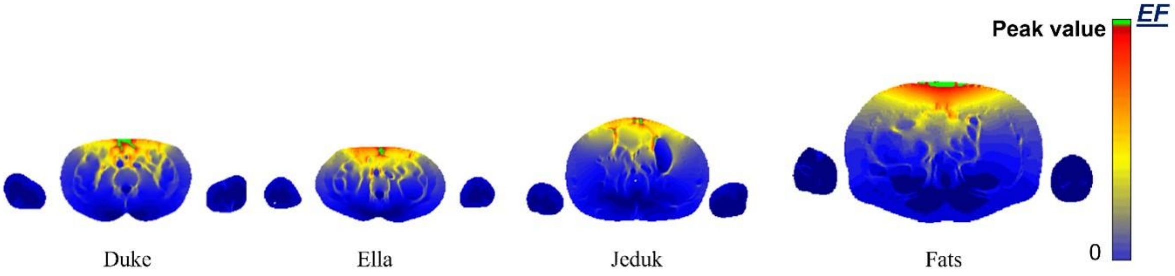

Supplementary Figure 4: Local EF for each model highlighted in light green: 5.5 V/m for Duke, 5 V/m for Ella, 5 V/m for Jeduk and 4.5 V/m for Fats.

Following is showed the chest exposure case, Supplementary Figure 5, in which are highlighted in light green the local peak of the induced EF observed in the transversal slice the height of the chest, directly exposed to the coil source.

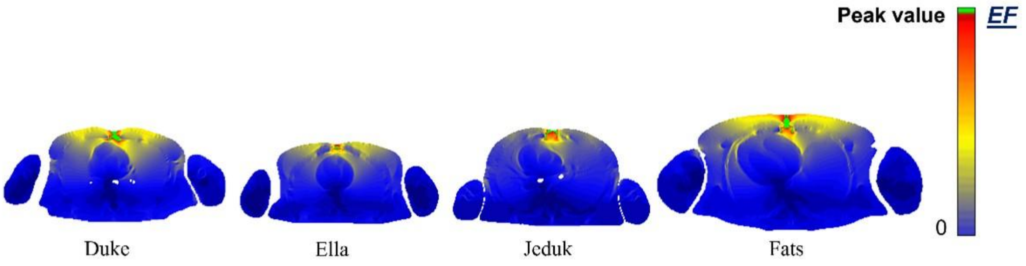

Supplementary Figure 5: Local EF for each model highlight in light green: 6 V/m for Duke, 8 V/m for Ella, 8.2 V/m for Jeduk and 6.5 V/m for Fats.

For what concerns the exposure condition involving the chest in specifically selected tissues (skin, SAT, fat, and breast) within a defined portion of the thoracic area (24 cm box). In Table *7* the computed 99th percentiles are reported, joined with the values induced only inside the SAT, that is the tissue in which we observed the higher values of the induced EF.

Table 7. 99th percentiles of the induced E-field (V/m) for selected tissues in the 24 cm box

Exposure *70% MSO*	**DUKE**	**ELLA**	**JEDUK**	**FATS**

**ABDOMEN**	*SAT*	5.89	4.38	5.61	4.01
	SAT + skin + Fat	5.79	4.46	5.06	3.9
**CHEST**	*SAT*	6.96	4.86	8.88	4.82
	SAT + skin + Fat + Breast	3.55	4.13	7.89	4.80

It is confirmed that with respect to the induced EF computed in the whole body, the percentiles found in the SAT tend to be higher. The 99th percentiles obtained from the analysis suggest an inverse relationship between the intensity of the induced E-field in the subcutaneous fat and its dimensions. This is evident from the lowest value observed in the Fats model. It is likely that a thinner SAT confines the EF to a smaller region, resulting in higher induced EF values. However, it is important to note that the values presented in Table 6, as they only pertain to specific tissues, cannot be used to draw conclusions regarding risk assessment.

4 Exposure to circular coil: trend of the induced electric field along the body of the subjects

Referring to the Figures 7-8, which shows the trend of the 99th percentile of the induced EF along the subjects' bodies, here it is shown how the results are obtained. Starting from the head of each subject, the induced EF was calculated in a box with a height of 10 cm (approximately fifty slices of tissue). Therefore, every point in the figure represents the 99th percentile of EF detected in each one box. The following are the details of the logic used to obtain these results.

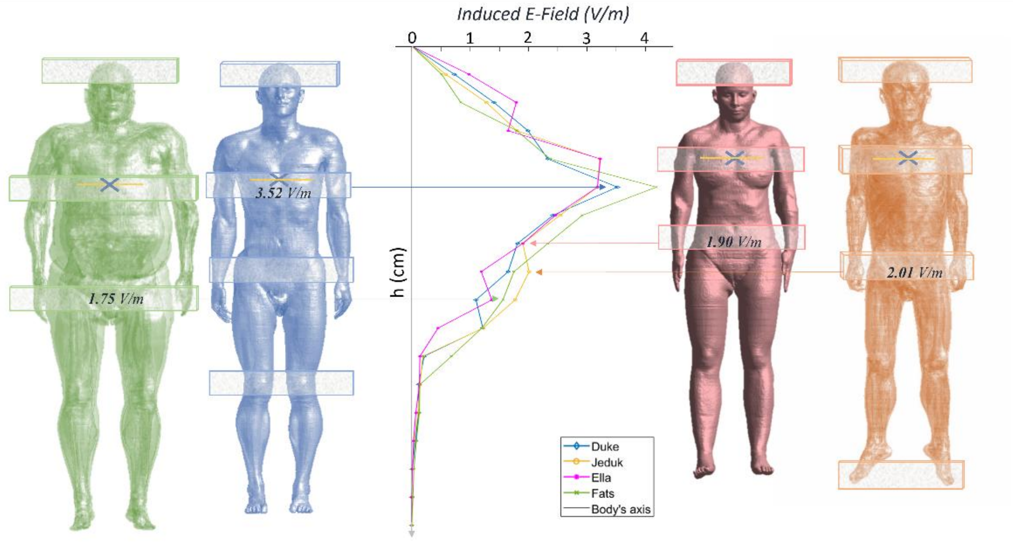

Supplementary 6. Exposure of the chest. Logic for the graph construction representing the trend of the induced electric field along the subjects' bodies**.**

The same logic was used to obtain the trend along the subjects' bodies in the case of abdomen exposure. As it is possible to see, the construction that starting from the head don't allow the centre the coil

perfectly in centre of a box, however it is expected and allow at the same time to appreciate the variation along the body of the induced quantity and the differences among the subjects.

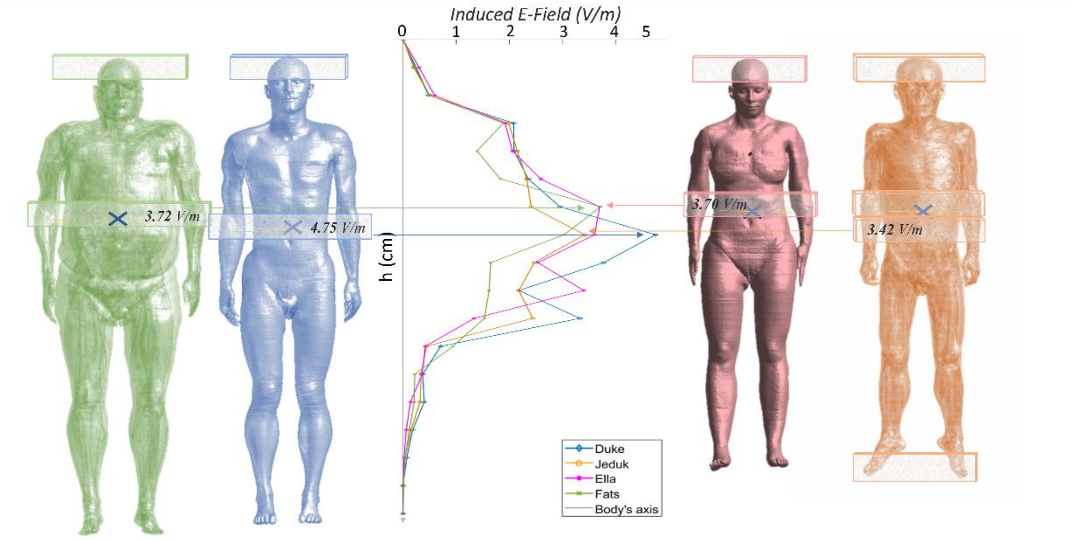

Supplementary Figure 7. Exposure of the abdomen. Logic for the graph construction representing the trend of the induced electric field along the subjects' bodies

As can be seen, for both exposure cases, the construction starting from the head does not, as we expected, allow the coil to be perfectly centred in the centre of the box. This, however, is not a problem from the point of view of the results, as the pattern allows us to appreciate the variation along the body of the induced quantity and the differences between the subjects at the same time.

5 Computational characteristics

Simulations were carried out on a high-performance workstation (AMD Ryzen 9 5950X, 16-Core Processor, 4.40 GHz, 128 GB RAM) using Sim4Life v7.0.
The computation time for each configuration ranged from approximately 30 minutes to 1 hour, depending on the specific exposure scenario.. No frequency-scaling approximation was applied; the excitation source was modeled as a pure sinusoidal current at the equivalent frequency of 1 kHz.

References

1. website. Physical Agents Portal. https://www.portaleagentifisici.it/index.php?lg=IT

2. Müller-dahlhaus F, Zipser CM, Premoli I, Kir J, Rossini P, Zrenner C, Ziemann U, Belardinelli P. Brain Stimulation Short-interval and long-interval intracortical inhibition of TMS-evoked EEG potentials. *Brain Stimul* (2018) 11:818–827. doi: 10.1016/j.brs.2018.03.008

3. Nelson AJ, Hoque T, Gunraj C, Ni Z, Chen R. Bi-directional interhemispheric inhibition during unimanual sustained contractions. (2009) 13:1–13. doi: 10.1186/1471-2202-10-31

4. Premoli I, Király J, Müller-Dahlhaus F, Zipser CM, Rossini P, Zrenner C, Ziemann U,

Belardinelli P. Short-interval and long-interval intracortical inhibition of TMS-evoked EEG potentials. *Brain Stimul* (2018) 11:818–827. doi: 10.1016/j.brs.2018.03.008

5. Bottauscio O, Zucca M, Chiampi M, Zilberti L. Evaluation of the Electric Field Induced in Transcranial Magnetic Stimulation Operators. *IEEE Trans Magn* (2016) 52:1–4. doi: 10.1109/TMAG.2015.2489561

6. Paffi A, Camera F, Carducci F, Rubino G, Tampieri P, Liberti M, Apollonio F. A Computational Model for Real-Time Calculation of Electric Field due to Transcranial Magnetic Stimulation in Clinics. *Int J Antennas Propag* (2015) 2015:1–11. doi: 10.1155/2015/976854

7. Petrov PI, Mandija S, Sommer IEC, van den Berg CAT, Neggers SFW. How much detail is needed in modeling a transcranial magnetic stimulation figure-8 coil: Measurements and brain simulations. *PLoS One* (2017) 12:e0178952. doi: 10.1371/journal.pone.0178952

8. Gomez-Tames J, Laakso I, Hirata A. Review on biophysical modelling and simulation studies for transcranial magnetic stimulation. *Phys Med Biol* (2020) 65:24TR03. doi: 10.1088/1361-6560/aba40d

9. Gomez LJ, Dannhauer M, Koponen LM, Peterchev A V. Conditions for numerically accurate TMS electric field simulation. *Brain Stimul* (2020) 13:157–166. doi: 10.1016/j.brs.2019.09.015

10. So PPM, Stuchly MA, Nyenhuis JA. Peripheral Nerve Stimulation by Gradient Switching Fields in Magnetic Resonance Imaging. *IEEE Trans Biomed Eng* (2004) 51:1907–1914. doi: 10.1109/TBME.2004.834251

11. ICNIRP. Guidelines for limiting exposure to time-varying electric and magnetic fields (1 Hz TO 100 kHz). *Health Phys* (2010) 99:818–836. doi: 10.1097/HP.0b013e3181f06c86

12. Reilly JP. Magnetic field excitation of peripheral nerves and the heart: a comparison of thresholds. *Med Biol Eng Comput* (1991) 29:571–579. doi: 10.1007/BF02446087

13. REILLY JP. Principles of Nerve and Heart Excitation by Time-varying Magnetic Fields. *Ann N Y Acad Sci* (1992) 649:96–117. doi: 10.1111/j.1749-6632.1992.tb49600.x

14. Reilly JP. *Applied Bioelectricity*. (1998). doi: 10.1007/978-1-4612-1664-3

15. Reilly JP, Diamant AM. Neuroelectric mechanisms applied to low frequency electric and magnetic field exposure guidelines - Part II: Non sinusoidal waveforms. *Health Phys* (2002) 83:356–365. doi: 10.1097/00004032-200209000-00005
